# Supplementary material for: Prevalence of Schistosomiasis and Soil-Transmitted Helminthiasis and Their Risk Factors: A Cross-Sectional Study in Itilima District, North-Western Tanzania
Source: Life (Basel). 2023 Dec 12;13(12):2333. doi: 10.3390/life13122333 (PMC10745067; doi:10.3390/life13122333)
Supplement: Supplementary file 1 [file life-13-02333-s001.zip › Supplementaries to ITNE baseline thesis/Supplementary 2_Prevalence of schools.docx]

Table S1. Prevalence of *S. haematobium* by school

| **S/no** | **Schools** | **Prevalence of S. haematobium** |
| --- | --- | --- |
| 1 | Budalabujiga A | 32.8% |
| 2 | Budalabujiga B | 33.3% |
| 3 | Bulolambeshi | 1.69% |
| 4 | Bumera | 0% |
| 5 | Dasina A | 1.69% |
| 6 | Dasina B | 0% |
| 7 | Gambasingu A | 8.3% |
| 8 | Gambasingu B | 28.3% |
| 9 | Gaswa | 22.9% |
| 10 | Habiya | 8.5% |
| 11 | Idoselo | 11.9% |
| 12 | Ikindilo | 11.7% |
| 13 | Ikungulipu A | 0% |
| 14 | Ikungulipu B | 0% |
| 15 | Inalo | 9.8% |
| 16 | Kabale | 13.8% |
| 17 | Kashishi | 43.5% |
| 18 | Kinang’weli | 13.3% |
| 19 | Lagangabilili | 1.9% |
| 20 | Laini B | 1.69% |
| 21 | Luguru | 5% |
| 22 | Lung’wa | 36.1% |
| 23 | Madilana | 0% |
| 24 | Mahembe | 3.5% |
| 25 | Mhunze | 0% |
| 26 | Mitobo | 31.7% |
| 27 | Musoma | 6.5% |
| 28 | Mwamapalala | 8.3% |
| 29 | Mwamigagani | 0% |
| 30 | Mwamtani | 16.7% |
| 31 | Mwamungesha | 0% |
| 32 | Mwanhunde | 9.8% |
| 33 | Mwanunui | 45.5% |
| 34 | Mwaogama | 0% |
| 35 | Mwaswale | 6.7% |
| 36 | Nanga A | 1.6% |
| 37 | Nanga B | 5% |
| 38 | Nangale | 0% |
| 39 | Ndoleleji | 8.7% |
| 40 | Ng’esha | 0% |
| 41 | Ng’wabuki | 0% |
| 42 | Ng’walali | 11.3% |
| 43 | Ng’walushu | 12.9% |
| 44 | Ngeme | 15% |
| 45 | Ngumo | 1.7% |
| 46 | Nhobora A | 6.8% |
| 47 | Nhobora B | 10% |
| 48 | Nkoma A | 0% |
| 49 | Nkuyu | 27% |
| 50 | Ntenga | 1.8% |
| 51 | Nyang’ombe | 0% |
| 52 | Sagata | 11.5% |
| 53 | Sawida A | 11.7% |
| 54 | Sawida B | 6.8% |
| 55 | Senani | 0% |
| 56 | Sunzula A | 0% |
| 57 | Sunzula B | 1.6% |
| 58 | Zagayu | 45.8% |
| 59 | Zanzui | 1.8% |
| 60 | Chinamili | 5% |
| 61 | Laini A | 0% |
| 62 | Mwanunho | 9.8% |
