# Supplementary material for: Prevalence of Schistosomiasis and Soil-Transmitted Helminthiasis and Their Risk Factors: A Cross-Sectional Study in Itilima District, North-Western Tanzania
Source: Life (Basel). 2023 Dec 12;13(12):2333. doi: 10.3390/life13122333 (PMC10745067; doi:10.3390/life13122333)
Supplement: Supplementary file 1 [file life-13-02333-s001.zip › Supplementaries to ITNE baseline thesis/Supplementary 3_Prevalence of villages.docx]

Table S2: Prevalence of Schistosoma *haematobium* by village

| **S/no** | **Village name** | **Prevalence (%)** |
| --- | --- | --- |
| 1 | Mwaswale | 0% |
| 2 | Nanga | 2.9% |
| 3 | Nangale | 5.9% |
| 4 | Ndoleleji | 4.2% |
| 5 | Ng’wabuki | 0% |
| 6 | Nhobora | 18% |
| 7 | Nkoma | 11.1% |
| 8 | Sagata | 9.8% |
| 9 | Sasago/Zagayu | 57.9% |
| 10 | Budalabujiga | 26.1% |
| 11 | Chinamili | 3.5% |
| 12 | Dasina | 0% |
| 13 | Habiya | 0% |
| 14 | Kinang’weli | 0% |
| 15 | Laini | 0% |
| 16 | Luguru | 12% |
| 17 | Mhunze | 0.6% |
| 18 | Mwamapalala | 0% |
| 19 | Mwamigagani | 12.2% |
